# Genome Reduction in Tetraploid Potato Reveals Genetic Load, Haplotype Variation, and Loci Associated With Agronomic Traits

**DOI:** 10.3389/fpls.2018.00944

**Published:** 2018-07-03

**Authors:** Norma C. Manrique-Carpintero, Joseph J. Coombs, Gina M. Pham, F. Parker E. Laimbeer, Guilherme T. Braz, Jiming Jiang, Richard E. Veilleux, C. Robin Buell, David S. Douches

**Affiliations:** ^1^Potato Breeding and Genetics Program, Department of Plant, Soil and Microbial Sciences, Michigan State University, East Lansing, MI, United States; ^2^Department of Plant Biology, Michigan State University, East Lansing, MI, United States; ^3^Department of Horticulture, Virginia Tech, Blacksburg, VA, United States; ^4^Department of Horticulture, Michigan State University, East Lansing, MI, United States; ^5^Plant Resilience Institute, Michigan State University, East Lansing, MI, United States

**Keywords:** haplotype, dihaploid, genetic load, complex traits, linkage map

## Abstract

The cultivated potato (*Solanum tuberosum*) has a complex genetic structure due to its autotetraploidy and vegetative propagation which leads to accumulation of mutations and a highly heterozygous genome. A high degree of heterozygosity has been considered to be the main driver of fitness and agronomic trait performance in potato improvement efforts, which is negatively impacted by genetic load. To understand the genetic landscape of cultivated potato, we constructed a gynogenic dihaploid (2*n* = 2*x* = 24) population from cv. Superior, prior to development of a high-density genetic map containing 12,753 single nucleotide polymorphisms (SNPs). Common quantitative trait loci (QTL) were identified for tuber traits, vigor and height on chromosomes 2, 4, 7, and 10, while specific QTL for number of inflorescences per plant, and tuber shape were present on chromosomes 4, 6, 10, and 11. Simplex rather than duplex loci were mainly associated with traits. In general, the Q allele (main effect) detected in one or two homologous chromosomes was associated with lower mean trait values suggesting the importance of dosage allelic effects, and the presence of up to two undesired alleles in the QTL region. Loss of heterozygosity has been associated with a lower rate of fitness, yet no correlation between the percent heterozygosity and increased fitness or agronomic performance was observed. Based upon linkage phase, we reconstructed the four homologous chromosome haplotypes of cv. Superior. revealing heterogeneity throughout the genome yet nearly duplicate haplotypes occurring among the homologs of particular chromosomes. These results suggest that the potentially deleterious mutations associated with genetic load in tetraploid potato could be mitigated by multiple loci which is consistent with the theory that epistasis complicates the identification of associations between markers and phenotypic performance.

## Introduction

Cultivated potato is an autotetraploid, highly heterozygous, and vegetatively propagated species. Tetrasomic inheritance comprises multiple genotypic configurations with up to four alleles and various combinations of alleles and dosage per locus. The more diverse alleles are at a locus, the greater the heterozygosity and number of allelic and epistatic interactions (Carputo and Frusciante, 2011). At any given locus of a tetraploid clone, there are up to three types of intra-locus interactions that could result in non-additive effects: first order (between two alleles), second order (among three alleles), and third order (among four alleles) while allelic dosage could mediate additive effects of intra-locus interactions. There may be more complexity for the optimal allelic combinations, locus interactions, and genetic effects when modeling quantitative traits. Elevated heterozygosity and genetic load have also been considered the main drivers of high and low vigor, respectively, associated with agronomic trait performance of cultivated potato. Inbreeding depression after self-pollination, and the superiority of tetraploid potato due to heterozygosity and polyploidy established a breeding bias toward increased heterotic diversity (De Jong and Rowe, 1971; Mendoza and Haynes, 1974). Besides the effects of epistasis, gain or loss of allelic diversity could be responsible for heterosis and inbreeding depression, respectively; recessive undesired alleles in the homozygous state would be expected to decrease fitness whereas allelic diversity at heterozygous loci facilitate both dominant and overdominant effects (Miranda Filho, 1999; Ceballos et al., 2015). Consistent with this hypothesis, a large-scale genome resequencing survey of genetic load in asexually propagated cassava revealed that the amount of deleterious mutations is greater in cultivated cassava compared to wild progenitors, as has been found in maize, sunflower and rice, and that cultivated cassava has a markedly greater number of mutations in the heterozygous rather than the homozygous state which could mask the lethal effects of recessive deleterious mutations (Ramu et al., 2017; Wang et al., 2017). The asexual propagation and polyploidy of cultivated potato give the potential of retaining greater mutational load, and also the generation of genome plasticity that enhances adaption to environmental changes. Copy number variation (CNV) in cultivated tetraploid potato is widely distributed throughout the genome and has been associated with lowly expressed genes and genes that respond to biotic and abiotic stress (Pham et al., 2017).

Dihaploids (2*n* = 2*x* = 24) from the cultivated tetraploid potato *Solanum tuberosum* (2*n* = 4*x* = 48) have been a valuable tool for genetic and cytogenetic studies as well as for breeding. Peloquin et al. (1991) reviewed the use of dihaploids to support evidence of tetrasomic inheritance, determine the basic chromosome number within the *Solanum* genus, discover meiotic mutations, understand ploidy and evolution, and assess sexual compatibility and hybridization barriers in potato. Dihaploid progeny of potato can be produced by anther culture or by chromosome elimination, sometimes referred to as “prickle pollination.” Specific haploid-inducer lines induce chromosome elimination; following fertilization from a cross of a tetraploid maternal clone with a haploid inducer, the paternal chromosomes are selectively eliminated from the developing hybrid embryo. By introduction of a homozygous, dominant embryo spot marker into haploid inducers, gynogenic dihaploid seed can be selected by the absence of the purple embryo spot visible on the hypocotyl of embryos or seedlings (Hermsen and Verdenius, 1973). As gametic genotypic representations of autotetraploid potato, dihaploid populations can facilitate determination of the complex genetic structure of cultivated potato. The reduced genome complexity of dihaploids enables simpler segregation ratios than tetraploids and a better understanding of the genetic factors controlling traits of interest. Several dihaploid populations have been used to decipher monogenic or polygenic inheritance and gene action effects associated with morphologic, agronomic and disease resistance traits (Cipar and Lawrence, 1972; Matsubayashi, 1979; De Maine, 1984; Pineda et al., 1993; Song et al., 2005; Velasquez et al., 2007). Unilateral (4*x* × 2*x*) and bilateral (2*x* × 2*x*) crosses using dihaploids have also served as a bridge to generate simpler and more efficient breeding schemes, overcome hybridization barriers, and achieve introgression of adaptive traits in the cultivated potato (Chase, 1963; Peloquin et al., 1991; Rokka, 2009).

The potato cultivar “Superior” was released in 1962 by the University of Wisconsin as a round white variety with scab resistance, and medium maturity (Rieman, 1962). Currently, it is grown in the USA and Canada as a fresh market variety. Dihaploid populations of a potato variety exhibit uniparental segregation following genome reduction. Using cv. Superior. as a model in our study, we generated a dihaploid population extracted from cv. Superior. to observe the effects of unmasking genetic load on different agronomic traits and elucidate main genomic regions associated with trait performance to understand the genetic complexity of tetraploid potato.

## Materials and methods

### Plant material

A gynogenic dihaploid (2*n* = 2*x* = 24) population of 95 individuals was created from *S. tuberosum* Group Tuberosum tetraploid cv. Superior. The *S. tuberosum* Group Phureja haploid inducer IVP101, homozygous dominant for an embryo seed spot marker, was used as the pollinator. Seeds lacking a purple spot were grown and leaf tissue from *in vitro* plantlets subjected to flow cytometry to identify dihaploids (Owen et al., 1988). Peaks were compared to known monoploid and diploid controls.

### Genotyping

DNA was isolated from leaf tissue of the “Superior” parent and 95 dihaploid gynogenic progeny and Illumina compatible paired end libraries were constructed as described previously (Hardigan et al., 2016). Libraries were skim-sequenced on the Illumina HiSeq 2000 platform at low coverage, with a theoretical approximation of 8x coverage of the genome, to identify single nucleotide polymorphic (SNP) segregating markers. Adapters and low quality bases were removed from the raw reads using Cutadapt v. 1.8.1 (Martin, 2011) and cleaned reads were aligned using BWA-MEM v. 0.7.11r1034 (Li, 2013) to the *S. tuberosum* Group Phureja DM 1-3 516 R44 reference genome v4.04 (Hardigan et al., 2016). Genotypes were called using the GATK Unified Genotyper (McKenna et al., 2010). Markers with unexpected segregation, distorted segregation (Chi-square threshold *P*-value <0.01), and singleton markers (SNPs without any duplicates) or markers with just one duplicate were removed. The remaining high quality markers were used for map construction after excluding duplicate co-segregating markers. Raw sequences are available in the National Center for Biotechnology Information Sequence Read Archive under BioProject ID PRJNA335821.

### Linkage map and quantitative trait locus analysis

The TetraploidSNPMap software for biallelic SNP markers (Hackett et al., 2017), informative for allele dosage in an autotetraploid species, was used to generate the genetic map and quantitative trait locus (QTL) analysis. As a unique parent population, only simplex (AAAB, ABBB) and duplex (AABB) marker configurations in “Superior” were segregating in the dihaploid population. The expected segregation for simplex markers in the diploid progeny corresponded to a 1:1 homozygous: heterozygous genotypic ratio (AA:AB, BB:AB), and for duplex markers to a 1:4:1 genotypic ratio (AA:AB:BB). However, four homologs per parental chromosome are segregating in this population. Thus, the segregation obtained in the dihaploid progeny fits the autotetraploid segregation for a cross with a null male parent for simplex (AAAB × AAAA, ABBB × BBBB) and duplex (AABB × AAAA, AABB × BBBB) markers. The marker configurations of the different genotypes were recoded according to TetraploidSNPMap code (AAAA = 0, AAAB = 1, AABB = 2, ABBB = 3, and BBBB = 4). For simplex segregation (AA:AB = AAAA:AAAB, BB:AB = BBBB:ABBB), genotypes were recoded as 0 and 1; while for duplex segregation (AA:AB:BB = AAAA:AABB:BBBB), genotypes were recoded as 0, 1, and 2.

The linkage map was constructed according to Hackett et al. (2013). The different mapping steps were implemented in TetraploidSNPMap: analysis of single marker segregation; cluster into linkage groups; estimation of recombination frequency and logarithm_10_ of the odds ratio for linkage (LOD score); and ordering and inference of SNP linkage phase (Hackett et al., 2017). A preliminary test of cluster of simplex SNPs was done using JoinMap 4.1 (Van Ooijen, 2006). Markers were coded for cross-pollinated population type (<lmxll>). This step allowed identification and exclusion of problematic markers that did not cluster as part of linkage groups. In the mapping process in TetraploidSNPMap, problematic and near duplicate markers were also detected and excluded; these mainly corresponded to outliers in the clustering and metric multidimensional scaling (MDS) ordering steps. A high concordance between genetic and physical maps has been reported for potato mapping populations (Felcher et al., 2012; Sharma et al., 2013). As a final quality control of the generated linkage maps, marker genetic positions (cM) were plotted against their physical positions (Mb) on each chromosome to generate MaryMaps (Chakravarti, 1991).

Square root transformation of phenotypic data was performed to improve the QTL detection. A QTL interval mapping analysis with a step size of 1 cM was done to identify QTL. A logarithm of the odds (LOD) threshold calculated based on a test of 500 permutations was used to detect significant marker associations. Next, the trait was modeled as an additive function of the QTL allele effect on each of eight homologous chromosomes (four for each parent). In addition to a full additive model, any of four different QTL simple models could be fit in this population with a single parent segregation. Simplex QTL model (Qqqq × qqqq), where the Q allele drives the main effect, duplex QTL (QQqq × qqqq) with additive effects of Q allele (the QTL genotypes qqqq, Qqqq, QQqq have means of m, m+Q, m+2Q), duplex QTL with non-additive effects of Q allele (the QTL genotypes qqqq, Qqqq, QQqq have different means m1, m2, m3), and duplex QTL with dominant effects of Q allele (two QTL genotypes qqqq, Q_qq mean categories). A QTL fit a simple model when the value of Schwarz Information Criterion (SIC) (Schwarz, 1978) was smaller than or close to the value of the full model, at least with a difference of 2 units from other simple models.

### Field evaluations

The dihaploid population was grown from greenhouse- or field-produced tubers at the Montcalm Research Center, Lakeview, MI (MRC) and the Botany and Plant Pathology Farm, East Lansing, MI (BPP) of Michigan State University over 2 years. In 2014, greenhouse-grown tubers were harvested in March and planted at MRC. In 2015, tubers produced in the field in 2014 in addition to greenhouse-grown tubers, harvested between February and March, were planted at MRC and BPP, respectively. In 2015, greenhouse tubers were subjected to a Rindite treatment to break dormancy prior to planting (Varga and Ferenczy, 1956). Thus, a total of three location/year datasets is reported in this study. All trials had a randomized complete block design using plots of eight plants as experimental unit and three replications per clone. Parents and progeny were evaluated for eight traits: total tuber yield (TTY) measured as g/plant, average tuber weight (ATW) in g, tuber set (TS) as number of tubers per plant, plant vigor (Vigor) scored as overall plant canopy development ~3 months after planting using a 1–5 scale (1: low vigor, 5: high vigor), plant height (Height) in cm assessed when plants started flowering, number of inflorescences per plant counted after a line initiated flowering in the plot (Infl/plant), specific gravity (SPGR) calculated using the formula [air weight/(air weight–water weight)] for a minimal sample size of 1 kg/plot, and tuber shape (Shape) scored using a 1–5 scale (1 = compressed, 2 = round, 3 = oval, 4 = oblong and 5 = long).

### Heritability and correlation analysis

The restricted maximum likelihood method (REML) was used to calculate broad-sense heritability (H^2^) with clones as random effects and site-year environments as random fixed effects. The heritability was estimated on a genotype mean basis as the ratio of:

$$\begin{array}{l} H^{2}=\frac{\sigma_{g}^{2}}{\left(\sigma_{g}^{2}+\frac{\sigma_{g*s-y}^{2}}{m}+\frac{\sigma_{e}^{2}}{rm}\right)} \end{array}$$

where $\left(\sigma_{g}^{2}\right)$, $\left(\frac{\sigma_{g*s-y}^{2}}{m}\right)$, and $\left(\frac{\left(\sigma_{e}^{2}\right)}{rm}\right)$ are the genetic, genotype × site-year environment interaction and residual variance components, m is the number of site-year environments and r is the number of replications.

Pearson correlation was used to estimate correlations between traits among site-year environments using the REML method when samples were missing. Means, variances, correlation and distribution analyses were calculated using JMP® 10 SAS Institute Inc., Cary, NC, USA.

### Fluorescence *in situ* hybridization

Chromosome preparation and fluorescence *in situ* hybridization (FISH) were performed using published protocols (Braz et al., 2018). Individual potato chromosomes of “Superior” were identified using two “barcode probes,” which contain 27,306 and 27,366 oligonucleotides (45 nt), respectively, derived from 26 different regions on the 12 potato chromosomes. These two probes produce 26 distinct FISH signals. Each of the 12 potato chromosomes is labeled with distinct signal pattern (Braz et al., 2018). FISH images were captured using a QImaging Retiga EXi Fast 1394 CCD camera and were processed with Meta Imaging Series 7.5 software. The final contrast of the images was processed using Adobe Photoshop CS3 software.

### Rescue of dwarf mutant with gibberellic acid treatment

Plantlets of the dihaploid VT_SUP_46 from the “Superior” dihaploid population maintained *in vitro*, were obtained after subculture on regular MS medium (Murashige and Skoog, 1962) (MS basal medium with vitamins + 3% sucrose + 0.6% plant agar micropropagation grade, pH 5.8, reagents from Phytotechnology Laboratories, Shawnee Mission, KS, USA). Assay tubes with plantlets were placed in a growth room at 22°C and 16-h photoperiod. Plantlets were grown in regular MS medium and medium supplemented with 0.3 mg/l of zeatin riboside (ZR, *trans* isomer, Sigma, St Louis MO USA) and two concentrations of gibberellic acid (GA_3_, Research Products International, Mt Prospect, IL, USA), 0.02 or 0.2 mg/l to rescue from the unique dwarf phenotype observed in this clone.

## Results

### Phenotypic performance

A total of 95 dihaploid clones was generated from crosses of cv. Superior. with IVP101; however, due to the low vigor of many of the dihaploids there was limited production of planting material in the greenhouse, and/or delayed emergence in the field such that between 50 and 75 individual clones were evaluated for the various traits under field conditions. For SPGR, 39 clones could be assessed in the MRC-2014 trial (Table 1). A wide range of variation of traits was observed in the population (Table 1). The quantile diagnostic plots showed a trend toward normal distribution for the population means of TS, Height, SPGR, and Shape, while bimodal normal distributions were observed for the means of TTY, ATW, Vigor and Infl/plant (Supplementary Figure 1). Transgressive segregation for TS, Vigor, Height, Infl/plant, and SPGR was detected in the progeny. For TTY and ATW, a few progeny performed similar to the parental line skewing the distribution toward greater values. Similarly, a few individuals with many Infl/plant skewed the distribution of this trait, especially in MRC-2014 and BPP-2015 site-year environments. Even though the normal distribution for SPGR was not affected by the skewness, there was tendency toward low values as shown by the negative skewness.

**Table 1:** Frequency distribution statistics of cv. Superior. and its dihaploid population (Pop) for 3 site-year environments [Montcalm Research Center (MRC) in 2014 and 2015, and Botany and Plant Pathology Farm (BPP) in 2015].

**Trait**	**Site-Year**	**Superior cv. Mean**	**Pop N**	**Pop Mean**	**Pop Std Dev**	**Pop Min**	**Pop Max**	**Pop Skewness**	**Pop % CV**	**Pop Kurtosis**	**% Inbreeding depression**
TTY	MRC-2014	605	59	135	124.09	7.13	579.88	1.418	92.25	1.99	77.8
	BPP-2015	1013	75	255	251.09	1.60	1119.75	1.634	98.65	2.64	74.9
	MRC-2015	921	58	260	190.05	0.00	781.54	0.897	73.17	0.35	71.8
ATW	MRC-2014	138	59	40	19.57	11.50	109.06	1.175	49.52	1.80	71.4
	BPP-2015	165	75	42	24.94	4.00	111.84	0.671	59.27	0.02	74.5
	MRC-2015	107	58	40	19.91	0.00	100.94	0.963	49.68	0.95	62.4
TS	MRC-2014	4.36	62	2.84	1.90	0.00	7.22	0.268	66.75	−0.80	34.8
	BPP-2015	6.14	75	5.02	3.24	0.36	13.40	0.726	64.57	0.12	18.2
	MRC-2015	8.67	58	6.15	3.35	0.00	13.59	0.267	54.46	−0.54	29.1
Height	MRC-2014	49.33	61	26.41	13.59	6.00	53.67	0.176	51.45	−1.07	46.5
	BPP-2015	56.50	74	35.18	14.57	9.00	71.33	0.229	41.42	−0.44	37.7
	MRC-2015	51.33	58	38.23	14.22	14.00	71.00	0.117	37.19	−0.80	25.5
Vigor	MRC-2014	4.33	61	2.62	1.19	1.00	5.00	0.053	45.45	−1.03	39.5
	BPP-2015	4.50	75	3.13	1.21	1.00	5.00	0.005	38.71	−1.12	30.5
	MRC-2015	5.00	58	3.49	1.35	1.00	5.00	−0.526	38.55	−1.10	30.2
Inf/plant	MRC-2014	1.67	50	1.40	1.28	0.00	6.00	2.273	91.42	6.05	16.2
	BPP-2015	2.00	69	2.86	2.43	0.30	10.67	1.629	84.96	2.03	−43.1
	MRC-2015	0.80	58	1.39	1.50	0.00	5.30	1.090	107.96	0.00	−73.2
SPGR	MRC-2014	1.07	39	1.07	0.01	1.05	1.10	−0.096	1.03	−0.16	−0.4
	BPP-2015	1.07	68	1.06	0.01	1.04	1.08	−0.442	0.96	0.16	0.9
	MRC-2015	1.08	54	1.07	0.01	1.04	1.09	−0.762	0.90	0.42	0.7
Shape	MRC-2014	2.83	60	2.86	0.87	1.50	5.00	0.769	30.29	−0.02	−1.0
	BPP-2015	2.50	75	3.08	0.56	2.00	4.67	0.401	18.10	−0.12	−23.3
	MRC-2015	2.17	57	2.83	0.73	1.67	4.83	0.910	25.91	0.43	−30.6

*Total tuber yield (TTY) in g/plant, average tuber weight (ATW) in g, tuber set (TS) as number of tubers per plant, plant height (Height) in cm, plant vigor (Vigor) 1: low vigor, 5: high vigor, number of inflorescences per plant (Infl/plant), specific gravity (SPGR), and tuber shape (Shape) 1 = compressed, 2 = round, 3 = oval, 4 = oblong and 5 = long*.

Overall, the number of days after planting required for 75% of plants per clone in a plot to emerge varied from 24 to 100 days. MRC-2014 had a significantly greater average number of days (63) to emerge compared with 52 and 42 days for BPP-2015 and MRC-2015, respectively (*P*-value <0.0001). The high correlation between MRC-2014 and BPP-2015 emergence data (0.53, *P*-value <0.0001), and low correlation of these locations with MRC-2015 (0.36 and 0.30, respectively; *P*-value <0.01 and 0.02, respectively) showed that the greenhouse-produced tubers used as planting material at both locations had similar longer emergence period compared to the field-grown tubers from the previous season that was used as seed for MRC-2015; this seed source appeared to be the main driver of more efficient emergence for most of the dihaploid clones (35.9 days for 75% of the population). The planting material did not have a critical effect on the reproducibility of data as the broad sense heritability was greater than 0.7 for all traits (Table 2). Comparison of correlations among data from different locations for the same trait, revealed that nearly all correlations were greater than 0.5 with *P*-values <0.0001. A low correlation was observed only for SPGR in BPP-2015 with MRC-2014 and MRC-2015 (0.24 and 0.4; *P*-values < 0.01 and 0.004, respectively), whereas the correlation between MRC-2014 and MRC-2015 for SPGR was 0.85 (*P*-value <0.0001). The low tuber yield for many individuals limited the total number of progeny evaluated for SPGR, therefore this trait was excluded from the QTL analysis.

**Table 2:** Heritability for eight agronomic traits evaluated in the “Superior” dihaploid population.

**Trait**	**Heritability (%)**
TTY	92.3
ATW	90.8
TS	86.9
Height	83.9
Vigor	89.0
Infl/plant	77.9
SPGR	75.8
Shape	88.6

*Total tuber yield (TTY) in g/plant, average tuber weight (ATW) in g, tuber set (TS) as number of tubers per plant, plant height (Height) in cm, plant vigor (Vigor) 1: low vigor, 5: high vigor, number of inflorescences per plant (Infl/plant), specific gravity (SPGR), and tuber shape (Shape) 1 = compressed, 2 = round, 3 = oval, 4 = oblong and 5 = long*.

High positive correlations among TTY, TS, ATW, Height, and Vigor were observed for all 3 site-year environments (Tables 3–5). Infl/plant showed high and moderate positive correlations for all 3 site-year locations. Tuber shape had low to no correlation with the other traits. The longer emergence period was highly correlated with low Height and Vigor for all three environments, while moderate to low negative correlations were observed between emergence and the three tuber traits, TTY, ATW, and TS.

**Table 3:**
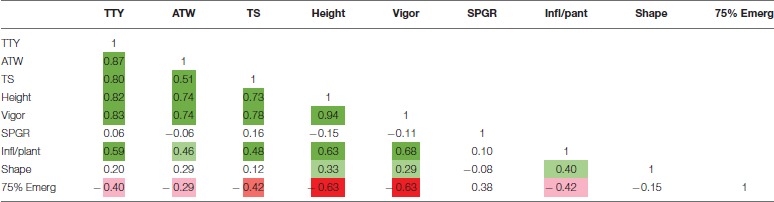
Correlation analysis for field season at Montcalm Research Center in 2014 for nine traits.

*Total tuber yield (TTY) in g/plant, average tuber weight (ATW) in g, tuber set (TS) as number of tubers per plant, plant height (Height) in cm, plant vigor (Vigor) 1: low vigor, 5: high vigor, number of inflorescences per plant (Infl/plant), specific gravity (SPGR), and tuber shape (Shape) 1 = compressed, 2 = round, 3 = oval, 4 = oblong and 5 = long, 75% of emergence number of days after planting (75% Emerg). Significant positive correlation dark green P-value < 0.0001, light green P-value < 0.05, significant negative correlation dark red P-value < 0.0001, intermediate red P-value < 0.001, and light red P-value < 0.001*.

**Table 4:**
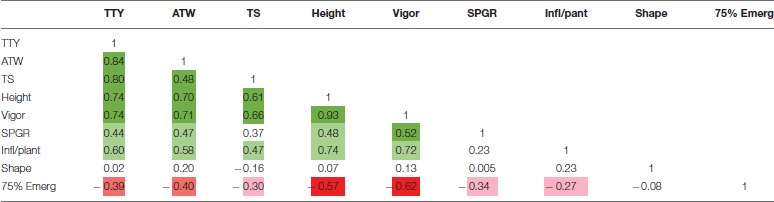
Correlation analysis for field season at Botany and Plant Pathology Farm in 2015 for nine traits.

*Total tuber yield (TTY) in g/plant, average tuber weight (ATW) in g, tuber set (TS) as number of tubers per plant, plant height (Height) in cm, plant vigor (Vigor) 1: low vigor, 5: high vigor, number of inflorescences per plant (Infl/plant), specific gravity (SPGR), and tuber shape (Shape) 1 = compressed, 2 = round, 3 = oval, 4 = oblong and 5 = long, 75% of emergence number of days after planting (75% Emerg). Significant positive correlation dark green P-value < 0.0001, light green P-value < 0.05, significant negative correlation dark red P-value < 0.0001, intermediate red P-value < 0.001, and light red P-value < 0.001*.

**Table 5:**
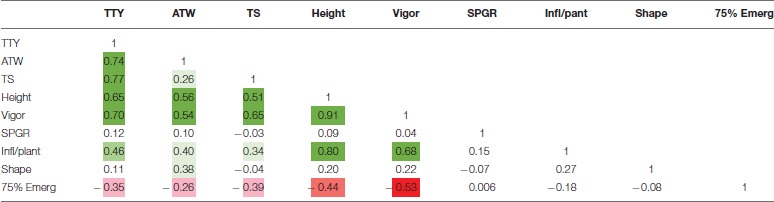
Correlation analysis for field season at Montcalm Research Center in 2015 for nine traits.

*Total tuber yield (TTY) in g/plant, average tuber weight (ATW) in g, tuber set (TS) as number of tubers per plant, plant height (Height) in cm, plant vigor (Vigor) 1: low vigor, 5: high vigor, number of inflorescences per plant (Infl/plant), specific gravity (SPGR), and tuber shape (Shape) 1 = compressed, 2 = round, 3 = oval, 4 = oblong and 5 = long, 75% of emergence number of days after planting (75% Emerg). Significant positive correlation dark green P-value < 0.0001, light green P-value < 0.05, significant negative correlation dark red P-value < 0.0001, intermediate red P-value < 0.001, and light red P-value < 0.001*.

A site-year environmental effect was detected for all traits except ATW. MRC-2015 reported significantly greater mean values and MRC-2014 the lowest for TS, Height, and Vigor, while BPP-2015 had the greatest values for Infl/plant and Shape, and BPP-2015 and MRC-2015 for TTY [*P*-values <0.001 for all except ATW (0.072) and Shape (0.008)]. For 53 dihaploid clones with full data, we detected significant (*P*-value <0.0001) genotype-environment interactions in all locations for TTY, TS, ATW, Vigor, Height, and Shape.

### Linkage map

A high-density genetic map was built for the 95-progeny of “Superior” dihaploid population. After filtering to identify high-quality segregating markers (Supplementary Table 1), we identified 12,753 polymorphic SNPs that were successfully mapped (Table 6 and Supplementary Table 2). The SNPs were located mainly at intergenic regions (10,159, 79.7%), compared to genic regions (2594, 20.3%), with 746 in exons and 1,970 in introns, and 120 overlapping both positions due to alternative splicing. The genetic map has a length of 1299.1 cM with 819 to 1374 SNPs per chromosome. The average inter-locus distance was 0.7 cM with a genome coverage of 99.3% relative to the 12 chromosomes in the current potato genome assembly.

**Table 6:** “Superior” linkage map length in centimorgans (cM), physical length in megabase pairs (Mb), and features of mapped single nucleotide polymorphisms (SNPs).

							**Interval distance cM**		
**Chr**	**Total SNP**	**N Seg**	**Bins**	**cM**	**Mb**	**PGSC v4.03 Mb**	**Min**	**Max**	**Mean**
chr01	1216	182	170	108.8	88.3	88.7	0.01	5.98	0.64
chr02	853	174	166	106.8	47.1	48.6	0.01	6.43	0.65
chr03	1235	169	165	106.4	62.01	62.3	0.01	4.89	0.65
chr04	1148	153	129	143.8	72.01	72.2	0.01	9.02	1.12
chr05	819	169	154	96.9	51.8	52.1	0.01	4.42	0.63
chr06	1246	164	158	92.5	58.9	59.5	0.01	3.95	0.59
chr07	1374	178	170	94.8	56.6	56.8	0.01	3.69	0.56
chr08	1201	139	139	90.3	56.7	56.9	0.01	5.37	0.65
chr09	847	149	143	104	61.3	61.5	0.01	4.73	0.73
chr10	978	119	109	91.4	59.5	59.8	0.01	5.43	0.85
chr11	897	134	116	159.8	45.2	45.5	0.01	19.88	1.39
chr12	939	210	187	103.7	60.8	61.2	0.01	6.12	0.56
Total	12753	1940	1806	1299.1	720.1	725.1	0.01	19.88	0.72

*Chromosome (Chr), potato genome sequence consortium assembly version 4.03 (PGSC v 4.03), unique segregating markers (Seg)*.

### QTL identified

**Figure 1:**
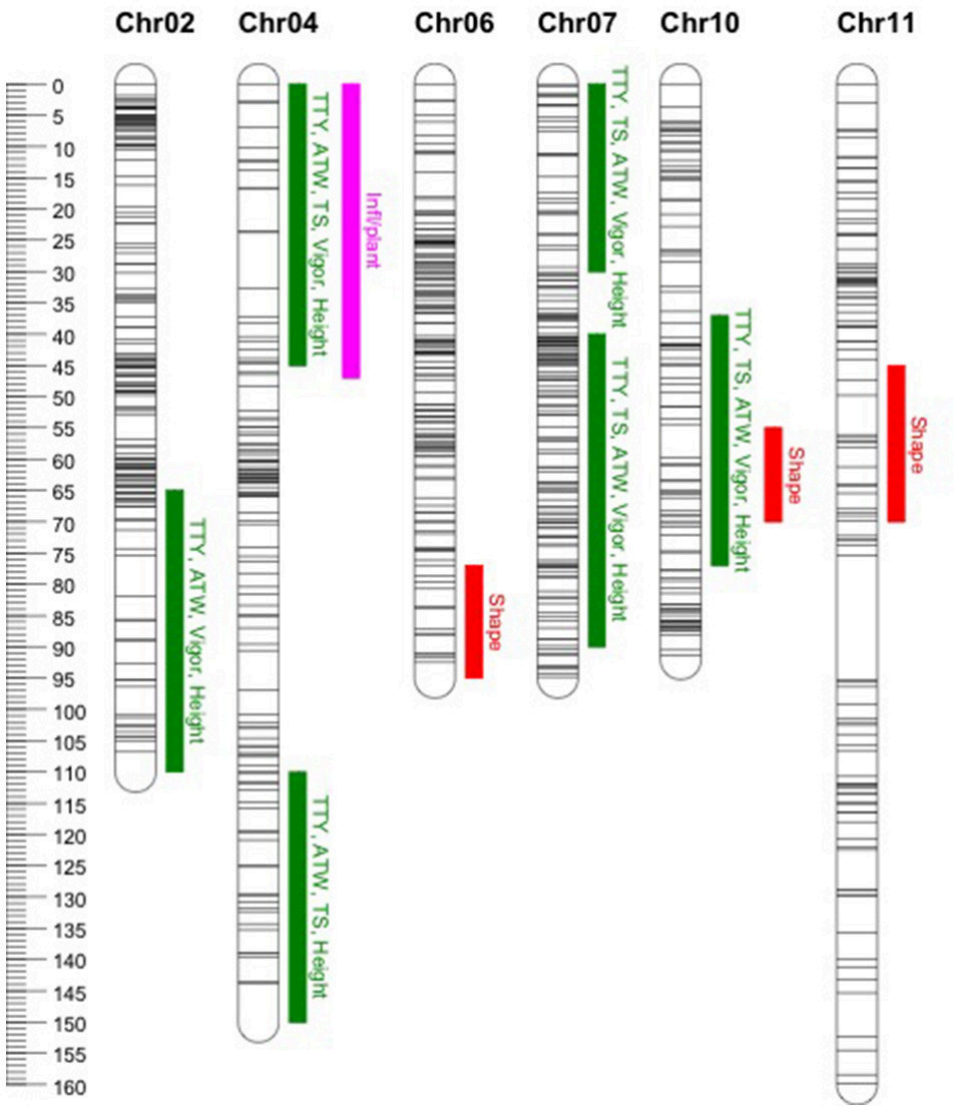
Location of identified quantitative trait loci (QTL). Common QTL as listed in Table 7 for total tuber yield (TTY), tuber set (TS), average tuber weight (ATW), Vigor and Height (green). Specific QTL for number of inflorescences per plant (Inf/plant) and tuber shape (Shape) in pink and red, respectively. On the left the cM genetic position scale for the mapped SNPs represented with horizontal lines along the chromosomes.

In general, common QTL were identified on chromosomes 2, 4, 7, and 10 for TTY, TS, ATW, Height, and Vigor, while specific QTL were identified for Infl/plant and Shape on chromosomes 4, 6, 10, and 11 (Figure 1). In some cases, the QTL were not identified in all site-year environments for each trait, as the peak was not always significant or not detected. Table 7 summarizes the QTL chromosome locations, phenotypic variation, QTL genetic model and homologous chromosomes associated with the Q allele effect. For most of the QTL, the closest SNPs to the QTL peak, co-segregating in phase with the Q alleles, were also reported.

**Table 7:** QTL identified in the “Superior” dihaploid mapping population, chromosome (Chr), and genetic centimorgan position (cM), logarithm of the odds (LOD) significance, variance explained (*R*^2^).

**Trait**	**QTL locus**	**Chr**	**cM**	**LOD**	**% R2**	**QTL genetic model**	**Homologous chromosome**	**Site-year location**	**Main Q effect**
TTY	chr02_47.91_11358	chr02	105	3.7	12.1	Simplex	H4	MRC-2014	↓
	chr02_47.47_d1954	chr02	102	2.6	6.2	Duplex, no additive	F14	MRC-2015	↓
	chr04_1.5_s247	chr04	9	2.5	7.7	Simplex	H2	MRC-2014	↓
	chr04_1.5_s247	chr04	10	3.1	10.1	Simplex	H2	BPP-2015	↓
	chr04_2.23_s502	chr04	16	3.0	11.8	Simplex	H2	MRC-2015	↓
	chr04_70.05_s12931	chr04	139	3.2	13.7	Simplex	H2	MRC-2014	↑
	chr07_1.77_s639	chr07	12	3.8	17.4	Simplex	H2	MRC-2014	↓
	chr07_1.77_s639	chr07	15	3.1	13.1	Simplex	H2	MRC-2015	↓
	chr07_52.52_s10951	chr07	76	2.8	9.5	Simplex	H1	MRC-2014	↓
		chr07	76	4.3	19.3	Full Model		MRC-2015	
	chr10_50.67_d1945	chr10	60	2.6	8.6	Duplex	F14/V14	MRC-2014	↑
	chr10_44.85_s7410	chr10	45	2.7	8.3	Simplex	H1	BPP-2015	↑
TS	chr04_1.5_s247	chr04	10	4.0	17.6	Simplex	H2	MRC-2014	↓
	chr04_1.5_s247	chr04	12	3.1	10.4	Simplex	H2	BPP-2015	↓
	chr04_2.23_s502	chr04	16	4.5	21.7	Simplex	H2	MRC-2015	↓
	chr04_70.05_s12931	chr04	143	2.8	10.2	Full Model^*^		MRC-2014	↑
	chr07_1.77_s639	chr07	12	2.7	9.8	Simplex	H2	MRC-2015	↓
	chr07_52.52_s10951	chr07	76	2.7	9.6	Simplex	H1	MRC-2015	↓
	chr10_46.48_s7781	chr10	57	2.3	4.7	Simplex	H1	MRC-2014	↑
		chr10	60	2.3	6.7	Full Model		MRC-2015	
ATW	chr02_47.91_11358	chr02	105	2.8	8.4	Simplex	H4	MRC-2014	↓
	chr02_47.47_d1954	chr02	93	3.1	11.7	Duplex, dominant	D23	MRC-2015	↑
	chr04_70.05_s12931	chr04	143	2.5	8.5	Simplex	H2	MRC-2014	↑
	chr04_1.5_s247	chr04	10	2.6	7.3	Simplex	H2	BPP-2015	↓
	chr07_1.77_s639	chr07	12	3.9	16.9	Simplex	H2	MRC-2014	↓
		chr07	76	2.6	8.7	Full Model		MRC-2014	
	chr10_50.49_d1897	chr10	65	2.2	6	Duplex, additive	V14	MRC-2014	↑
	chr10_44.85_s7410	chr10	45	3.9	14.4	Simplex	H1	BPP-2015	↑
Vigor	chr02_47.91_11358	chr02	105	4.2	14.5	Simplex	H4	MRC-2014	↓
	chr02_46.24_10991	chr02	96	2.6	6.4	Simplex	H4	BPP-2015	↓
	chr04_1.5_s247	chr04	10	4.8	22.3	Simplex	H2	MRC-2014	↓
	chr04_0.02_d8	chr04	10	2.7	7.8	Duplex, additive	V13	BPP-2015	↑
	chr04_1.5_s247	chr04	10	3.4	14.6	Simplex	H2	MRC-2015	↓
	chr07_1.77_s639	chr07	14	2.9	11.5	Simplex	H2	MRC-2015	↓
		chr07	76	2.6	8.6	Duplex, no additive	F14	MRC-2014	
		chr07	76	2.9	11.1	Duplex, no additive	F14	MRC-2015	
	chr10_44.85_s7410	chr10	45	2.7	8.2	Simplex	H1	BPP-2015	↑
Height	chr02_47.91_11358	chr02	106	3.4	10.4	Simplex	H4	MRC-2014	↓
	chr02_46.24_10991	chr02	96	3.0	8.3	Simplex	H4	BPP-2015	↓
	chr04_1.5_s247	chr04	10	4.6	20.9	Simplex	H2	MRC-2014	↓
	chr04_0.02_d8	chr04	10	2.7	7.4	Duplex, additive	V13	BPP-2015	↑
	chr04_2.23_s502	chr04	16	3.3	13.4	Simplex	H2	MRC-2015	↓
	chr04_70.05_s12931	chr04	139	2.3	6.8	Simplex	H2	MRC-2014	↑
	chr07_1.77_s639	chr07	14	2.6	8.5	Simplex	H2	MRC-2015	↓
		chr07	76	2.5	7.8	Duplex, no additive	F14	MRC-2014	
		chr07	76	2.3	6.7	Duplex, no additive	F14	MRC-2015	
	chr10_44.85_s7410	chr10	47	2.6	9.2	Simplex	H1	MRC-2014	↑
	chr10_44.85_s7410	chr10	45	2.3	5.7	Simplex	H1	BPP-2015	↑
Infl/plant	chr04_1.91_s379	chr04	10	3.5	13.3	Simplex	H3	BPP-2015	↑
	chr04_1.91_s379	chr04	10	2.6	8.5	Simplex	H3	MRC-2015	↑
Shape		chr06	93	2.5	7.9			MRC-2014	
		chr06	93	2.4	7.6	Duplex, no additive	F13	MRC-2015	
		chr10	60	5.1	18.6	Duplex, no additive	F13	MRC-2014	
		chr10	64	3.1	8.3	Duplex, no additive	F13	BPP-2015	
		chr10	60	4.3	15.5	Duplex, no additive		MRC-2015	
	chr11_42.28_s7725	chr11	142	3.5	14.8	Simplex	H2	MRC-2014	↓
	chr11_43.16_d2698	chr11	130	2.8	7.8	Duplex, no additive	F12	BPP-2015	↓
	chr11_42.28_s7725	chr11	135	4.4	21.5	Simplex	H2	MRC-2015	↓

*Total tuber yield (TTY) in g/plant, average tuber weight (ATW) in g, tuber set (TS) as number of tubers per plant, plant height (Height) in cm, plant vigor (Vigor) 1: low vigor, 5: high vigor, number of inflorescences per plant (Infl/plant), specific gravity (SPGR), and tuber shape (Shape) 1 = compressed, 2 = round, 3 = oval, 4 = oblong and 5 = long, main Q allele effect associated with lower (↓) or greater (↑) mean trait values*.

* *Significant marker even though a specific model was not detected*.

### Fluorescence *in situ* hybridization

**Figure 2:**
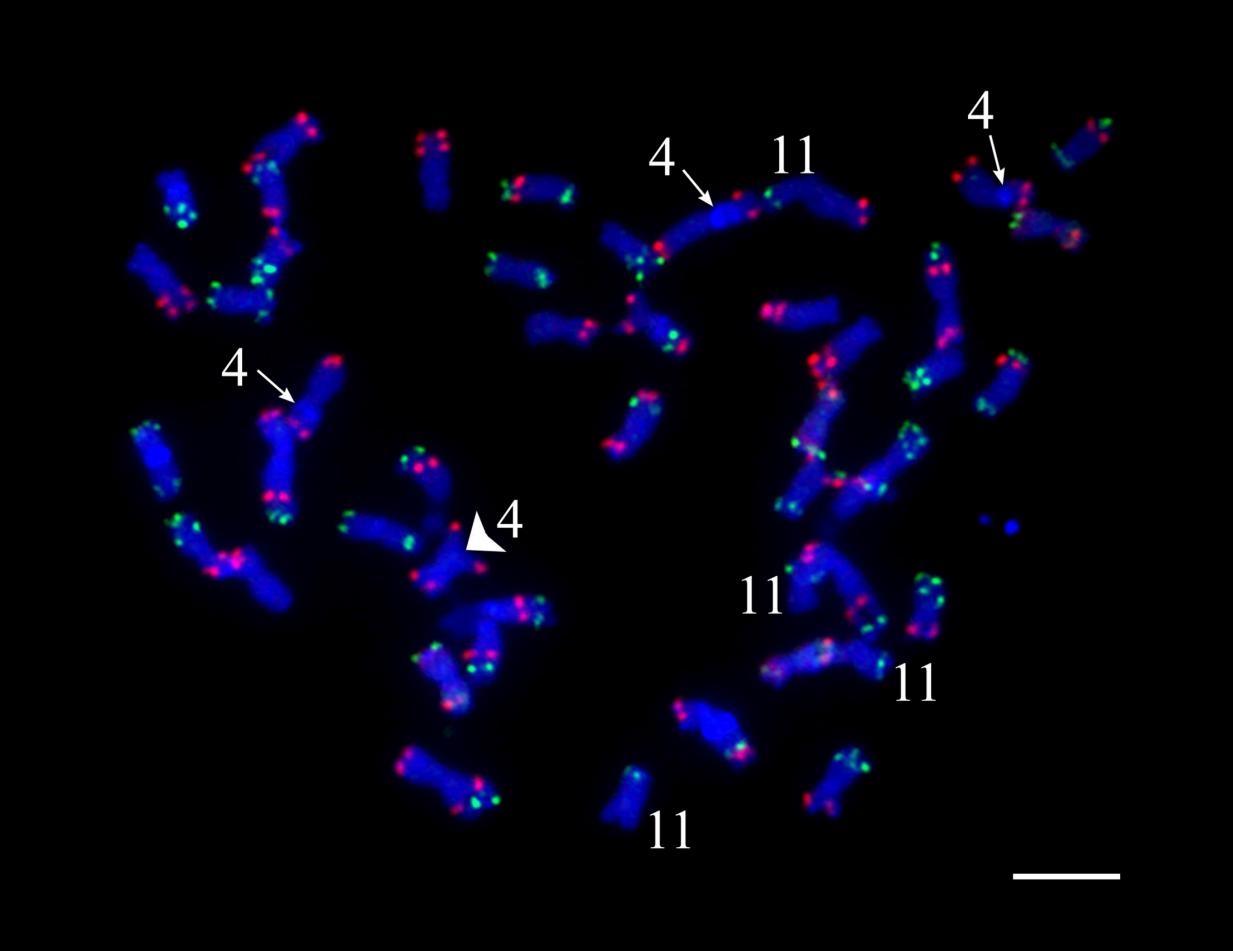
Fluorescent *in situ* hybridization visualization of cv. Superior. chromosomes. The knobs on three copies of chromosome 4 are indicated by an arrow, the knob-less copy of chromosome 4 is indicated by a large arrowhead. Four copies of chromosome 11 are also indicated.

Even though a strict quality filtering process was used in the selection of markers for linkage mapping, the construction of genetic maps for chromosomes 4 and 11 was especially problematic. We conducted oligo-based fluorescence *in situ* hybridization (Oligo-FISH) (Braz et al., 2018) to examine if the four copies of chromosomes 4 and 11 in “Superior” show visible structural variation. All 48 chromosomes could be individually identified based on the Oligo-FISH signal patterns (Figure 2). We did not observe any unambiguous chromosome structural changes associated with chromosomes 4 and 11. However, three copies of chromosome 4 contain a visible heterochromatic knob in the short arm, whereas the remaining copy of chromosome 4 does not contain the knob (Figure 2).

### Double reduction leads to a dwarf mutant

**Figure 3:**
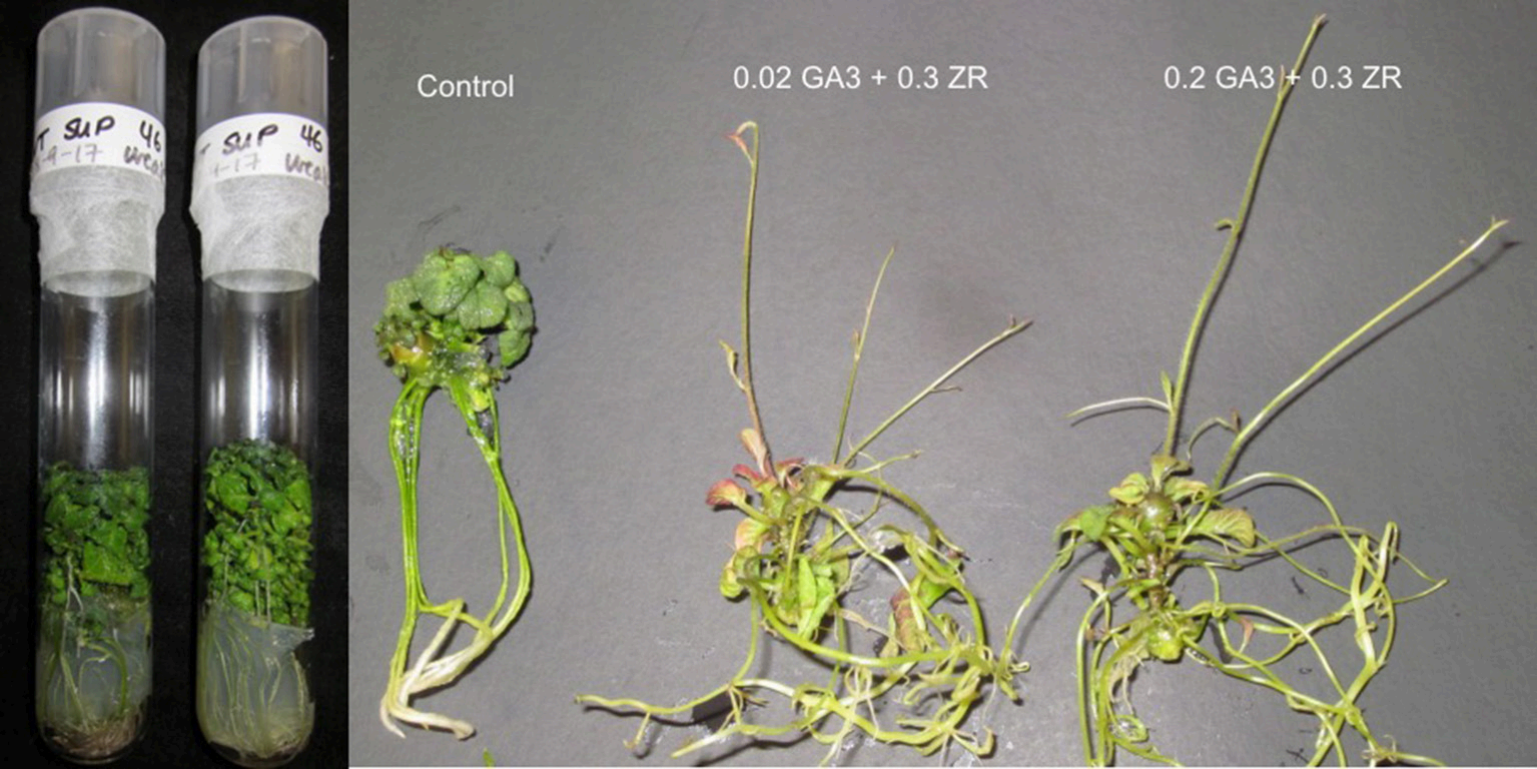
Gibberellic acid treatment recovers a normal phenotype in the dwarf dihaploid VT_SUP_46. Comparison of plant growth in regular propagation medium (control), with medium supplemented with (0.02 mg/l gibberellic acid-GA_3_ and 0.3 mg/l of zeatin riboside-ZR or 0.2 mg/l GA_3_ and 0.3 mg/l ZR).

A dark green and rosette dwarf phenotype that can be rescued by GA_3_ application has been reported in hybrid progeny of cv. Superior. as well as some other potato diploid and tetraploid clones (Bamberg and Hanneman, 1991; Valkonen et al., 1999). A single dihaploid, VT_SUP_46, within our “Superior” dihaploid population has a strong dwarf phenotype (Figure 3). Treatment of *in vitro* plantlets of VT_SUP_46 on propagation medium supplemented with GA_3_ (0.02 and 0.2 mg/l) resulted in rescue from the dwarf phenotype (Figure 3).

## Discussion

### Genetic load unmasked in cv. superior. dihaploid population

**Figure 4:**
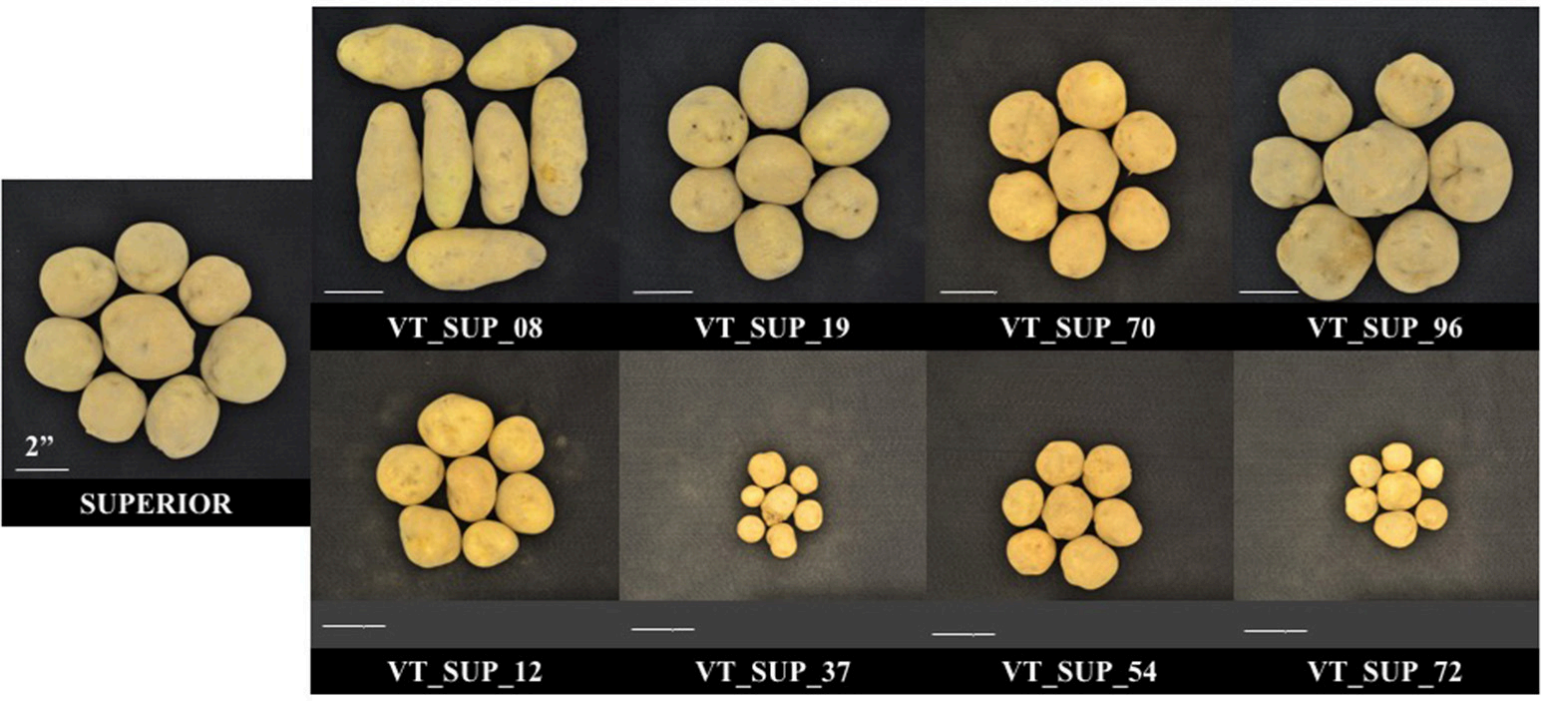
Phenotypic differences in tuber size and shape between cv. Superior. and its dihaploid progeny with high **(Top)** and low agronomic performance **(Bottom)** under field season at Montcalm Research Center in 2015.

As reported previously (Peloquin and Hougas, 1960; De Maine, 1984; Kotch et al., 1992; Hutten et al., 1995), segregation of a tetraploid parent configuration in a gametic dihaploid population leads to breakdown of allelic combinations and interactions, and to unmasking of the genetic load due to homozygosity of recessive alleles and/or the effects of dysfunctional alleles. A dihaploid population has an expected reduction of heterozygosity equivalent to three generations of self-pollination of an autotetraploid, which increases the probability of a homozygous state of recessive and deleterious alleles (Peloquin and Hougas, 1960). The effect of homozygous recessive and sub-lethal alleles in a duplex configuration in a locus in the parental line will lead to 17% weakness or loss in the progeny, to 50% when in a triple dose in a triplex parent genotype, and would not be detected in a simplex configuration (Hutten et al., 1995). In complex traits, several genes and their contribution to the genetic structure of the trait will influence the magnitude of the effect of recessive or sub-lethal alleles, producing a wide range of variation in phenotype. Hutten et al. (1995) evaluated 31 different dihaploid populations reporting some levels of dwarfism, wide variation between populations in the rate of tuberization ability, and low frequencies of flowering and pollen stainability. In fact, low fitness phenotypes prevented 35.8% (MRC-2014), 22.1% (BPP-2015), and 40% (MRC-2015) of the “Superior” dihaploid population to be evaluated under different site-years. Almost 20% of “Superior” dihaploids were never evaluated in the field due to extremely low vigor (Figure 4).

Using the theory proposed by Fasoulas (1988) where greater genetic load affecting a trait would increase the coefficient of variation (%CV), causing negative kurtosis and positively skewing the trait frequency distribution of a dihaploid population, Kotch et al. (1992) studied the frequency distribution statistics of several dihaploid potato populations. Skewness, kurtosis, and the inbreeding depression coefficient (relative percentage of dihaploid population mean compared to tetraploid parent mean) were used to indicate the type of gene action affecting different traits. In general, a negligible or low inbreeding depression coefficient, close to zero or minor skewness, negative or zero kurtosis, and low %CV indicated that the 4*x* parent had genes with primarily additive effects and low genetic load associated with the trait. In contrast, significant positive skewness, positive kurtosis, high %CV, and a high inbreeding depression coefficient are suggestive that the 4*x* parent primarily has genes with non-additive effects associated with the trait. In this “Superior” dihaploid population (Table 1), SPGR is potentially a trait mainly governed by genetic factors with additive effects and low genetic load, while for TTY and ATW genes that have non-additive effects and greater genetic load are suggested. For traits where the distribution statistics fit in the middle of these parameters, both additive and non-additive genetic effects could be mediating the phenotype, this is the case for TS, Height, Vigor, and Infl/plant.

By comparing the performance of different populations, Kotch et al. (1992) highlighted that a trait with similar %CV could have different inbreeding depression coefficients, which implies the importance of non-additive gene control rather than genetic load (fixation of deleterious genes). Evaluations of trait performance in inbred generations and diallelic crosses of outcrossing species (e.g., maize, cassava) suggest that the relevance of non-additive effects increases with the genetic complexity of a trait, and that a strong inbreeding depression effect will also be associated (Ceballos et al., 2015). Non-additive effects driving heterosis (dominance, overdominance, and epistasis) are particularly important for grain yield and fresh root/tuber yield. A similar complexity is suggested in potato yield traits, such as tuber yield with strong inbreeding depression as described in this analysis and reported in populations of self-pollinated tetraploids (Golmirzaie et al., 1998).

### Genome heterogeneity of cv. superior

A high-density genetic linkage map was built for the 95-progeny of “Superior” dihaploid population. For several chromosomes, it was difficult to order and estimate the linkage phase of the markers, particularly for chromosomes 4 and 11. With the MDS ordering approach (Preedy and Hackett, 2016), a continuous curve plot is expected because of the low linkage between markers located at opposite chromosome ends. The MDS graph showed sub-clusters of markers producing extra sub-curves within the general curve or outlier points in some instances. Excluding problematic markers solved this problem. Primarily genotype errors or distorted segregation could affect the marker quality and mapping process. This is not the case in our population since besides the threshold (*P*-value < 0.01) used to eliminate markers with distorted segregation, we did not detect any pattern with meaningful distorted segregation that could limit transmission of specific genomic regions. However, inversions in some homologs or structural variation between homologous chromosomes could also be associated with problems during linkage mapping. In fact, an inversion resulted in a large gap on chromosome 11, while on chromosome 4 we observed a tendency of independent clustering and mapping of the homologous chromosomes. Both chromosomes 4 and 11 showed a greater length than normally reported in previous diploid and tetraploid linkage maps (Hackett et al., 2013; Sharma et al., 2013; Manrique-Carpintero et al., 2015; Massa et al., 2015; Da Silva et al., 2017). For chromosome 4, this may be due to the large heterochromatic knob on three of the four homologous chromosomes (Figure 2).

**Figure 5:**
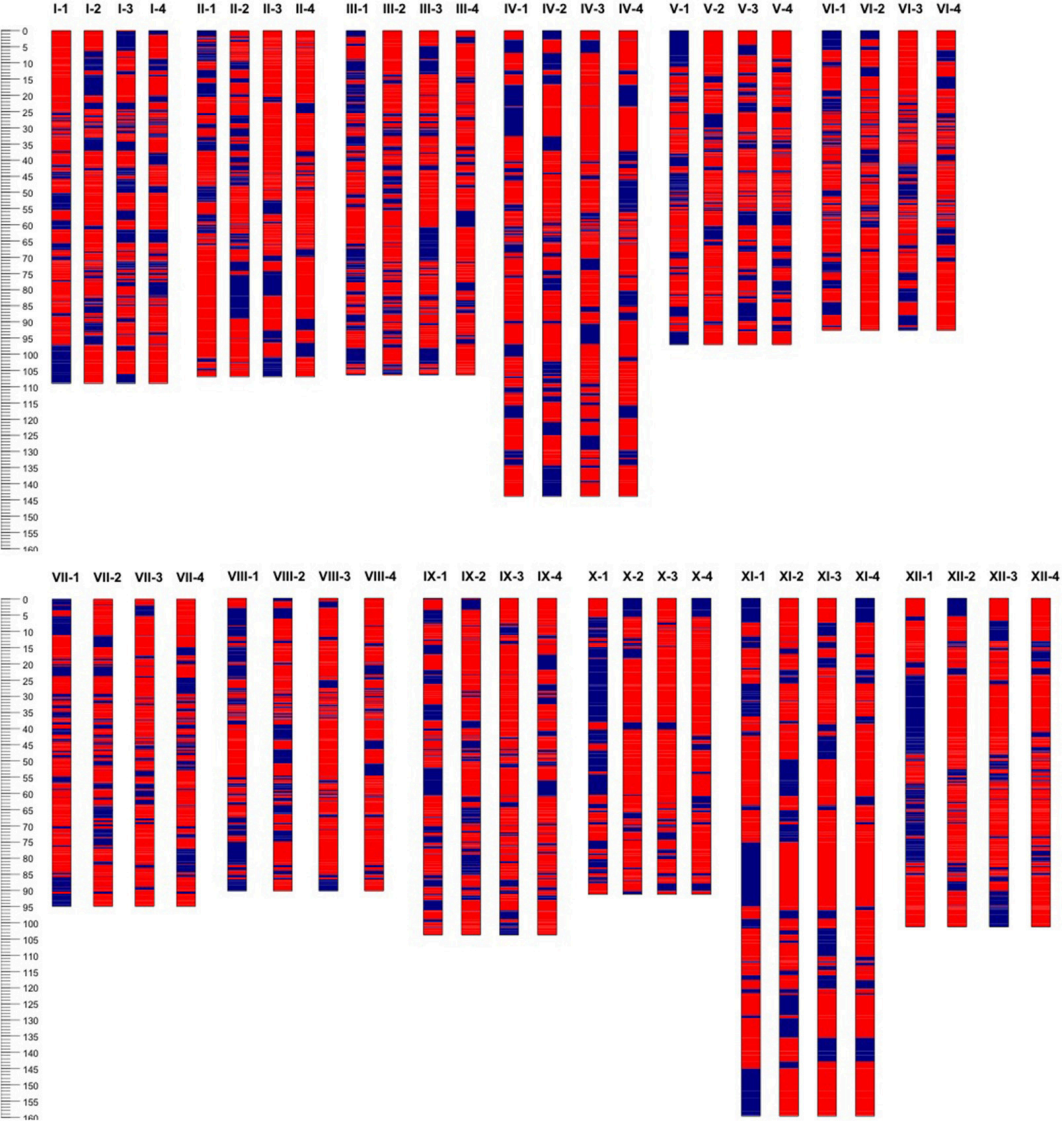
Haplotype configuration of four homologous chromosomes (1–4) or each of 12 (I–XII) chromosomes of cv. Superior. using unique segregating single nucleotide polymorphic (SNP) bi-allelic (A, B, red and blue respectively) markers.

Based on the linkage phase generated in the mapping process, we reconstructed the four haplotypes for each of the 12 homologous chromosomes of the “Superior” parent for the mapped loci (Figure 5). Then genetic distances were calculated between different pairs of homologs per chromosome using GGT 2.0 software (Van Berloo, 2008). Different patterns of differentiation among homologs per chromosome were observed based on the simple matching coefficient (the number of shared alleles as proportion of all alleles) distance measurement (Table 8). For instance, for chromosomes 10 and 12, only one homolog was markedly different from the other three, while for chromosomes 1, 2, and 5, a pair of homologous chromosomes were highly similar and the other types of homologs were distant. This analysis revealed a novel observation of a high level of heterogeneity among homologous chromosomes in a tetraploid potato cultivar.

**Table 8:**
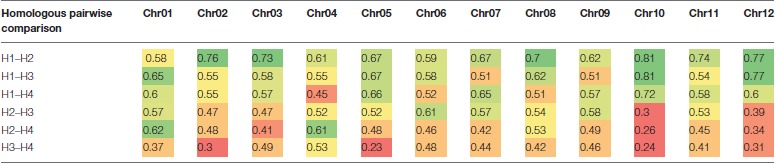
Genetic distance between homologs (H) of each chromosome (Chr) of cv. Superior.

*Distance color code: shorter genetic distance toward red while greater genetic distance greener*.

### QTL analysis of agronomic traits

Highly correlated traits shared QTL with similar positions and effects. For most of the QTL (chromosome 2, 4, and 7) for TTY, ATW, TS, Height, and Vigor, the Q allele was in simplex configuration and associated with lower trait mean values in heterozygous genotypes. When the Q allele was detected on two homologous chromosomes, the presence of any or both Q alleles was associated with lower mean values. This resulted in having a marker segregation in which 50 or 16.7% of the evaluated population showed lower fitness phenotype with the Q allele associated. This could be explained by the importance of dosage allelic effect in the genotype configuration of the tetraploid parent or that the tetraploid parent has mainly one and up to two weak or dysfunctional alleles in the QTL regions. Nevertheless, if the recessive detrimental alleles are in simplex configuration we do not expect homozygous allelic states unless double reduction has occurred. In contrast, for the QTL on chromosome 10, the Q alleles in heterozygous genotypes were associated with greater mean values of these traits. Based on the analysis of the statistics of distribution of phenotypic data, dominance, intra-locus interactions, and epistatic interaction effects were considered as the main types of gene action associated with TTY and ATW, while a combination of additive, dominance, intra-locus interactions, and epistatic interaction effects was evident for TS, Height and Vigor. Either additive or dominant effects could explain the QTL with simplex allelic effects detected for most of the traits, while the duplex QTL effects were explained by dominant, additive and interaction effects.

We did not find any specific QTL for TTY and ATW, the traits with the greatest inbreeding depression. We hypothesize that probably multiple loci with a low percentage of explained variance as well as their epistatic interactions could be the reason underlying a lack of power to detect these QTL. Similarly, major QTL may not be segregating in this specific population. A clear example is the maturity locus on chromosome 5 associated with *Dof Zinc Finger Protein*-*StCDF* gene (Kloosterman et al., 2013). We did not identify a QTL in that region even though three alleles for cv. Superior were reported by Hardigan et al. (2017). The “Superior” alleles have polymorphisms (non-synonymous SNPs and truncations) compared to the allele associated with short day tuberization photoperiod control *CDF* in *Solanum tuberosum* Group Andigena. Therefore, all of these alleles should have similar additive effects in which any combination of those alleles in the diploid progeny is not associated with a segregating phenotype. Infl/plant corresponded to a trait for which we observed no inbreeding depression. The statistics of distribution analysis suggested that this trait should have gene actions associated with additive effects. Simplex and duplex with no additive allelic effects were the main type of gene action identified in the QTL analysis. Considering that several loci contribute to the genetic structure of a quantitative trait, we expect that epistatic interactions may play a major role in the genetic structure of the evaluated traits (best allelic combinations at different loci). In fact, only a few individuals in the progeny reached a genetic structure that generated a phenotype similar to the tetraploid parent.

The common QTLs for TTY, ATW, TS, Vigor and Height on chromosomes 2, 4, 7, and 10 co-localized with previous QTL reported for one or a few of the evaluated agronomic traits. Interestingly, the single parent tetraploid segregation revealed that the QTL were collectively associated with all of these traits. A QTL on chromosome 2 was reported for TTY and tuberization (Van den Berg et al., 1996; McCord et al., 2011; Manrique-Carpintero et al., 2015), on chromosome 4 for ATW, tuber size and tuberization (Van den Berg et al., 1996; D'hoop et al., 2014; Manrique-Carpintero et al., 2015), on chromosome 7 for tuber yield (Schäfer-Pregl et al., 1998), and on 10 for tuber yield, tuber set, and Vigor (Schäfer-Pregl et al., 1998; Manrique-Carpintero et al., 2015; Rak et al., 2017). Bonierbale et al. (1993) reported QTL on chromosomes 2, 4, and 7 for TTY, TS, and ATW, although the QTL on chromosome 2 does not match the chromosome arm location of our QTL. Similarly, several authors have reported a QTL on chromosome 10 for tuber shape (Van Eck et al., 1994; Prashar et al., 2014; Lindqvist-Kreuze et al., 2015), separating mainly compressed, round, and oval from the more elongated shape types oblong and long.

### Importance of inbreeding

Loss of heterozygosity has been associated with lower fitness. Considering that the homozygous alleles in the “Superior” parent were also homozygous in the progeny, we tested if the amount of segregating heterozygosity inherited from the tetraploid parent was associated with any trait. There was no correlation for most of the traits and poor correlation between the percentage of inherited heterozygosity and increasing TTY and TS trait values for all 3 site-years (*R*^2^ = 0.07–0.09 and *P*-value < 0.03). As reported by Bonierbale et al. (1993), the additivity of a certain number of heterozygous loci rather than total heterozygosity makes a greater contribution to overall trait performance, along with the dominant alleles and epistatic effects. For instance, the weakest dihaploid clone (VT_SUP_46) had greater inherited heterozygosity than a high vigor and high-yielding dihaploid (VT_SUP_19), 60 and 55%, respectively. Haplotype analysis of “Superior” chromosomes showed a high level of heterogeneity in the parental genome. Cross-pollinated mating type, vegetative propagation, and polyploidy of cultivated potato contribute to retention of greater mutational load that is further complicated by rampant structural variation throughout the genome (Pham et al., 2017). Genetic load due to deleterious allelic mutations in the simplex configuration could be compensated by the alternative allele, but also by multiple loci with similar function(s) in the polyploid genome. At a given locus, it is possible to have: (i) duplicate alleles or alleles with synonymous nucleotide polymorphisms that will not affect the functionality at the protein level, (ii) alleles with polymorphisms that alter functionality at the protein level, and /or (iii) alleles with no functionality (i.e., a null allele). In principle, any alternative functional allele would compensate for dysfunctionality in a dihaploid or tetraploid individual when present with the lethal allele, at the same or different locus. Therefore, the combination of alleles at multiple loci determines the trait phenotype. However, epistasis complicates the identification of associations between markers and phenotypic performance (Ceballos et al., 2015). Inbreeding can be the most efficient method to organize the genome to combine favorable alleles interacting in a stable epistatic system, therefore high fitness progeny would have the best genetic structure (Jansky et al., 2016). By design, we examined only biallelic SNPs, thereby disregarding the contributions of multiallelic loci on yield attributing traits. For triallelic loci, 1/6th of the dihaploid progeny would be expected to be homozygous at any given SNP site whereas for tetraallelic loci, all dihaploid progeny would remain heterozygous, albeit with different combinations of alleles.

### Candidate genes

In the common QTL regions identified in this study (Figure 1) for TTY, ATW, TS, Height and Vigor, we hypothesized that candidate genes associated with overall plant growth and development, as well as tuberization (Supplementary Table 3) would be present. Hormonal regulation, sucrose metabolism, photoperiod, circadian clock, and age-dependent signaling pathways are involved in tuber initiation and growth (Navarro et al., 2015) for which some genes have been identified. In the QTL region on chromosomes 2 and 7, candidate genes in the photoperiod regulatory pathway associated with length of plant cycle and tuberization were identified (*Dof Zinc Finger Protein*-*StCDF3, CONSTANTS-CO*, and mi*RNA156*) around 46 and 2 Mb, respectively. High accumulation of sucrose and starch in terminal sink organs is enhanced by efflux from the leaves promoting tuberization, down-regulation of the phloem *Sucrose transporter 4 (SUT4)* gene is critical to the switch from apoplastic to symplastic phloem uploading (Chincinska et al., 2013). *SUT4* follows a circadian expression pattern, has reciprocal regulation with gibberellic acid (GA), and affects the expression of circadian-regulated genes, flowering, tuberization and shade avoidance. *SUT4* is located at 65.8 Mb on chromosome 4, in the region where a QTL was detected. The breakdown of active GA is required for tuberization and gibberellin 2-oxidase genes are part of the mechanism that controls endogenous levels of GA (Kloosterman et al., 2007); we identified a *Gibberellin 2-oxidase 2 (GA2ox2)* candidate gene at 51.9 Mb in a QTL regions on chromosome 7. Interestingly, in the other QTL region of chromosome 7 at 1.9 Mb is a *Trehalose-phosphate synthase 1* (TPS1) gene with a potential role in the T6P regulatory pathway that was recently associated with flowering and tuberization in potato (Seibert et al., 2017). Ectopic expression of *Lonely Guy 1* (*LOG1*), a cytokinin-activating enzyme, drove the formation of aerial minitubers in tomato (Eviatar-Ribak et al., 2013). The plants displayed a unique transcriptome signaling network probably associated with the appropriated local hormonal balance for tuber formation. Differential expression and pleiotropic effects of *LOG* genes showed their major role in cytokinin metabolism to modulate plant growth and development in *Arabidopsis thaliana* (Kuroha et al., 2009). A cytokinin riboside 5′-monophosphate phosphoribohydrolase *LOG3* gene is located in the QTL region at 56 Mb on chromosome 10. For tuber shape, several candidate genes associated with cell structure and function, and pectin metabolism have been reported in the major QTL located around 48 Mb on chromosome 10 (Lindqvist-Kreuze et al., 2015). Similarly, in the QTL region discovered in our analysis on chromosome 6, a *Pectinesterase* gene is located at 58 Mb.

### Dwarf phenotype

There is strong evidence that a dwarf phenotype observed in our “Superior” dihaploid population is the result of GA_3_ deficiency. The dark green and rosette dwarf phenotype has been reported in potato in hybrid progeny of cv. Superior. as well as some other potato diploid and tetraploid clones (Bamberg and Hanneman, 1991; Valkonen et al., 1999). In all cases, reversion of the dwarf phenotype occurred following GA_3_ application. A single recessive locus encoding *ga1* was proposed to cause the dwarf phenotype, which was confirmed by evaluation of test segregation in several crosses (Bamberg and Miller, 2012). The study also revealed that a gibberellin deficiency allele was in simplex configuration (GGGg) in “Superior.” The homozygous state gg of the recessive allele of a simplex locus in a dihaploid population is expected only due to double reduction, therefore a small proportion of dwarf phenotype would be observed in the dihaploid progeny. In fact, VT_SUP_46 is a unique clone in our “Superior” dihaploid population with a strong dwarf phenotype. Examination of the regions with potential double reduction in VT_SUP_46, revealed the end of chromosomes 6 as the candidate region. However, a few other clones also showed double reduction but did not have dwarf phenotype, suggesting that other loci could compensate the GA_3_ supply in those dihaploid clones. When *in vitro* plantlets of VT_SUP_46 were grown on propagation medium supplemented with GA (0.02 and 0.2 mg/l) the plants elongated to a normal phenotype (Figure 3).

## Conclusion

Genetic load in the “Superior” cultivar was unmasked through the generation of a dihaploid population. The segregation of the parental tetraploid configuration identified major QTL regions associated with most of the evaluated agronomic traits. Interestingly, four chromosomes were identified with common QTL that could elucidate interconnected metabolism. Candidate genes regulating plant development and tuberization were identified in the QTL regions. Complementation of gene function due to homozygous deleterious alleles could play a major role in trait performance in polyploid potato.

## Author contributions

RV, CRB, and DD planned and designed the project. NM-C drafted the manuscript. JC and NM-C conducted phenotypic data analysis, linkage mapping, and QTL analysis. NM-C tissue culture experiment. GB and JJ made cytogenetic analysis. DD and JC were involved field experiments. GP generate genotypic data. RV and FL generated the Superior dihaploid population. All authors contributed to the editing of the manuscript and approved the final draft.

### Conflict of interest statement

The authors declare that the research was conducted in the absence of any commercial or financial relationships that could be construed as a potential conflict of interest.
