# Supplementary material for: Genome Reduction in Tetraploid Potato Reveals Genetic Load, Haplotype Variation, and Loci Associated With Agronomic Traits
Source: Front Plant Sci. 2018 Jul 3;9:944. doi: 10.3389/fpls.2018.00944 (PMC6037889; doi:10.3389/fpls.2018.00944)
Supplement: Supplementary file 1 [file Table_1.DOCX]

Supplementary Material

**Genome reduction in tetraploid potato reveals genetic load, haplotype variation, and loci associated with agronomic traits**

**Norma C. Manrique-Carpintero^1^, Joseph J. Coombs^1^, Gina Pham_2_, F. Parker E. Laimbeer^3^, Guilherme T. Braz^2^, Jiming Jiang^2,4^, Richard E. Veilleux^3^, C. Robin Buell^2, 5^, and David S. Douches^1*^**

*** Correspondence:**David S. Douches
douchesd@msu.edu

# Supplementary Tables

**Supplementary Table 1**. Filtering process to identify high-quality segregating single nucleotide polymorphic (SNP) markers.

| **Removed** | **Remaining** | **Filtering Step** |
| --- | --- | --- |
| **Simplex** |  |  |
|  | 152484 | Total number of simplex SNPs |
| 4786 | 147698 | Removed SNPs with unexpected segregation >= 10 (10% of 95 individuals) |
| 21716 | 125982 | Removed SNPs with chi-square P-value <0.01 |
| 85672 | 40310 | Removed singletons (SNPs without any duplicate) |
| 13756 | 26554 | Removed SNPs with just 1 co-segregating marker |
|  |  |  |
| **Duplex** |  |  |
|  | 87479 | Total number of duplex SNPs |
| 223 | 87256 | Removed SNPs with unexpected segregation >= 10 (10% of 95 individuals) |
| 28682 | 58574 | Removed SNPs with chi-square P-value <0.01 |
| 45969 | 12605 | Removed singletons (SNPs without any duplicate) |
| 6086 | 6519 | Removed SNPs with just 1 co-segregating marker |
|  |  |  |
|  | 33073 | Total number of markers for mapping |
| 20320 | 12753 | Removed in the mapping process |
