# Supplementary material for: Genome Reduction in Tetraploid Potato Reveals Genetic Load, Haplotype Variation, and Loci Associated With Agronomic Traits
Source: Front Plant Sci. 2018 Jul 3;9:944. doi: 10.3389/fpls.2018.00944 (PMC6037889; doi:10.3389/fpls.2018.00944)
Supplement: Supplementary file 3 [file Table_3.DOCX]

Supplementary Material

**Genome reduction in tetraploid potato reveals genetic load, haplotype variation, and loci associated with agronomic traits**

**Norma C. Manrique-Carpintero^1^, Joseph J. Coombs^1^, Gina Pham_2_, F. Parker E. Laimbeer^3^, Guilherme T. Braz^2^, Jiming Jiang^2,4^, Richard E. Veilleux^3^, C. Robin Buell^2, 5^, and David S. Douches^1*^**

*** Correspondence:**David S. Douches
douchesd@msu.edu

# Supplementary Tables

**Supplementary Table 3**. Candidate genes identified in the QTL regions, location in the potato genome sequence assembly 4.03, chromosome (Chr) and mega base pair (Mb).

| **Gene annotation** | **Locus acronym** | **Chr** | **Mb** | **Gene ID** |
| --- | --- | --- | --- | --- |
| Dof Zinc finger protein | *StCDF3* | chr02 | 46.1 | PGSC0003DMG400001330 |
| CONSTANT | *CO* | chr02 | 45.1 | PGSC0003DMG402010056 |
| Sucrose transporter 4 | *StSUT4* | chr04 | 65.8 | No annotated |
| Pectinesterase |  | ch06 | 59.5 | PGSC0003DMG400024049 |
| miRNA156 |  | chr07 | 0.6 | PGSC0003DMG400011134 |
| Trehalose-6-phosphate synthase | *TPS1* | chr07 | 1.9 | PGSC0003DMG400027449 |
| CONSTANT | *CO* | chr07 | 2.3 | PGSC0003DMG400027475 |
| Gibberellin 2-oxidase 2 | *GA2ox2* | chr07 | 51.9 | PGSC0003DMG400033046 |
| Cytokinin riboside 5'-monophosphate phosphoribohydrolase | *LOG3* | chr10 | 56 | PGSC0003DMG400028154 |
