# Supplementary material for: Genome Reduction in Tetraploid Potato Reveals Genetic Load, Haplotype Variation, and Loci Associated With Agronomic Traits
Source: Front Plant Sci. 2018 Jul 3;9:944. doi: 10.3389/fpls.2018.00944 (PMC6037889; doi:10.3389/fpls.2018.00944)
Supplement: Supplementary file 4 [file Image_1.PDF]

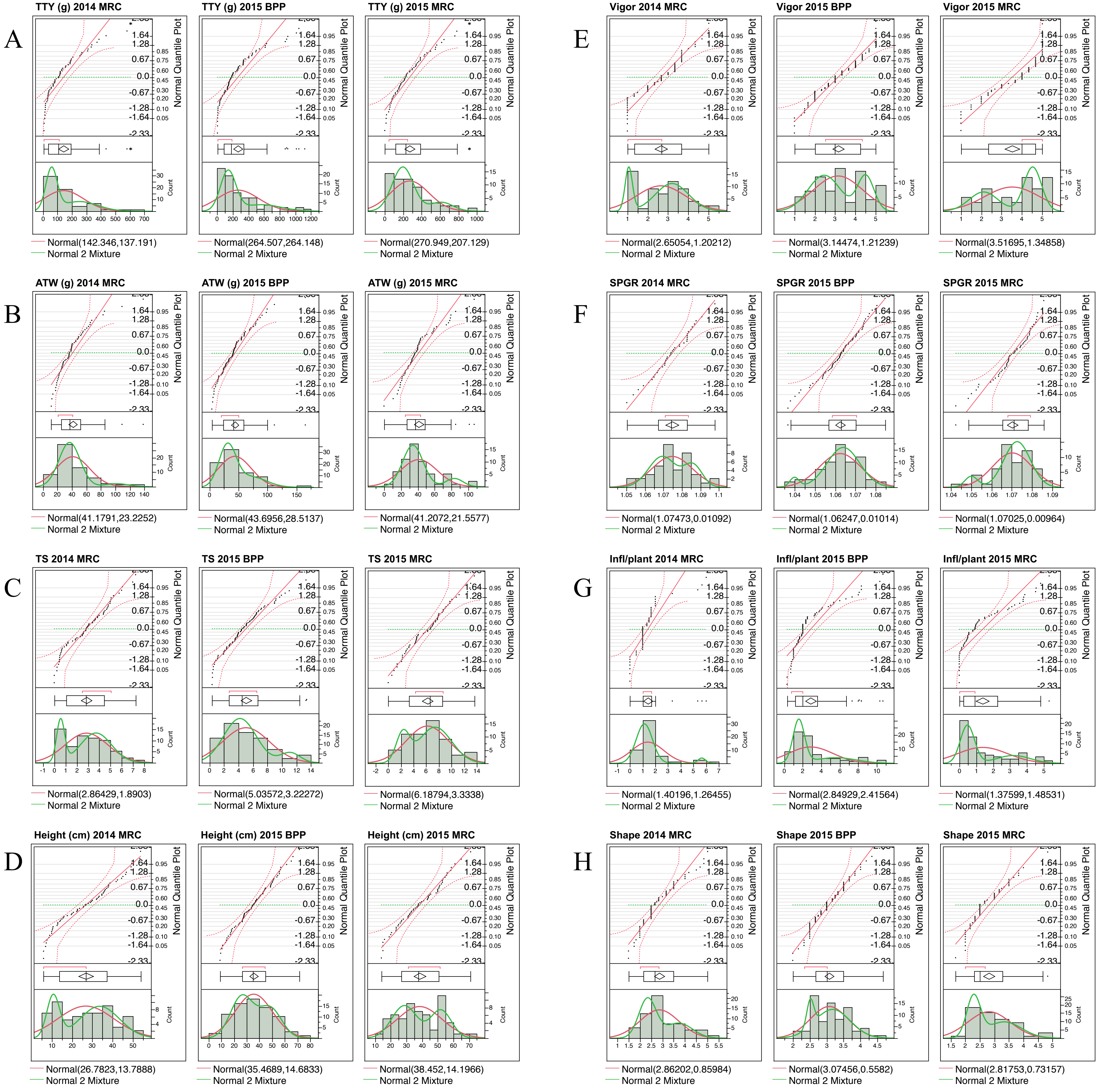

Supplementary Figure 1. Distribution of data represented by normal quantile plot (top), outlier box plot (middle) and histogram (bottom) for three site-year environments (Montcalm Research Center (MRC) in 2014 and 2015 , and Botany and Plant Pathology Farm (BPP) in 2015) and eight traits: A. Total tuber yield (TTY) in g/plant, B. average tuber weight (ATW) in g, C. tuber set (TS) as number of tubers per plant, D. plant height (Height) in cm, E. plant vigor (Vigor) 1: low vigor, 5: high vigor, F. specific gravity (SPGR), G. number of inflorescences per plant (Infl/plant), and H. tuber shape (Shape) 1=compressed, 2=round, 3= oval, 4=oblong and 5= long.
